# The Role of Decorin in the Ocular Surface: Structure, Function, and Therapeutic Potential

**DOI:** 10.1002/pgr2.70034

**Published:** 2025-07-22

**Authors:** Kartik Goel, Anil Tiwari, Shruti Rathore, Swati Sood, Virender S. Sangwan, Rajiv R. Mohan

**Affiliations:** 1 Departments of Ophthalmology, College of Veterinary Medicine and School of Medicine, University of Missouri, Columbia, Missouri, USA; 2 Eicher-Shroff Centre for Stem cells research (ESC SCR), Dr. Shroff Charity Eye Hospital, Delhi, India; 3 Medical Microbiology and Infectious Diseases, University of Manitoba, Manitoba, Canada; 4 Harry S. Truman Memorial Veterans’ Hospital, Columbia, Missouri, USA; 5 Mason Eye Institute, School of Medicine, University of Missouri, Columbia, Missouri, USA

## Abstract

The ocular surface, comprising the cornea, conjunctiva, and sclera, relies on a complex extracellular matrix (ECM) for structural support and functional integrity. Decorin (DCN), a small leucine-rich proteoglycan, a key ECM component involved in collagen fibrillogenesis, growth factor modulation, and cellular regulation, is essential for maintaining corneal transparency, wound healing, and preventing pathological fibrosis. DCN interacts with various target molecules, including transforming growth factor-beta (TGF-β), vascular endothelial growth factor (VEGF), and epidermal growth factor receptor (EGFR), influencing critical processes, such as cell proliferation, inflammation, and angiogenesis. These interactions underscore DCN’s therapeutic potential in ocular disorders like corneal scarring, pterygium, and keratoconus. This review explores the structure, expression, and functions of DCN in the ocular surface, emphasizing its regulatory mechanisms and therapeutic potential. Recent advancements include gene therapy approaches, sustained release systems, and topical applications, each demonstrating significant promise in treating ocular surface diseases. Additionally, the review highlights the importance of DCN in ocular disorders, including congenital stromal corneal dystrophy (CSCD), KC, and pterygium. This review aims to elucidate the multifaceted roles of DCN, paving the way for innovative therapeutic strategies in ocular surface therapy. Future directions for DCN research involve advanced delivery mechanisms, gene editing technologies, and expanded studies on other ocular surfaces.

## 1 | Introduction

The ocular surface, comprising the cornea, conjunctiva, and sclera, plays a critical role in maintaining vision and protecting the eye from environmental insults. The extracellular matrix (ECM) of the ocular surface is a complex network of proteins and proteoglycans that provides structural support, mediates cell-matrix interactions, and regulates cellular functions [1]. Among the key components of the ECM is Decorin (DCN), a small leucine-rich proteoglycan (SLRP) that has garnered significant attention for its diverse biological functions [2, 3].

**FIGURE 1 |:**
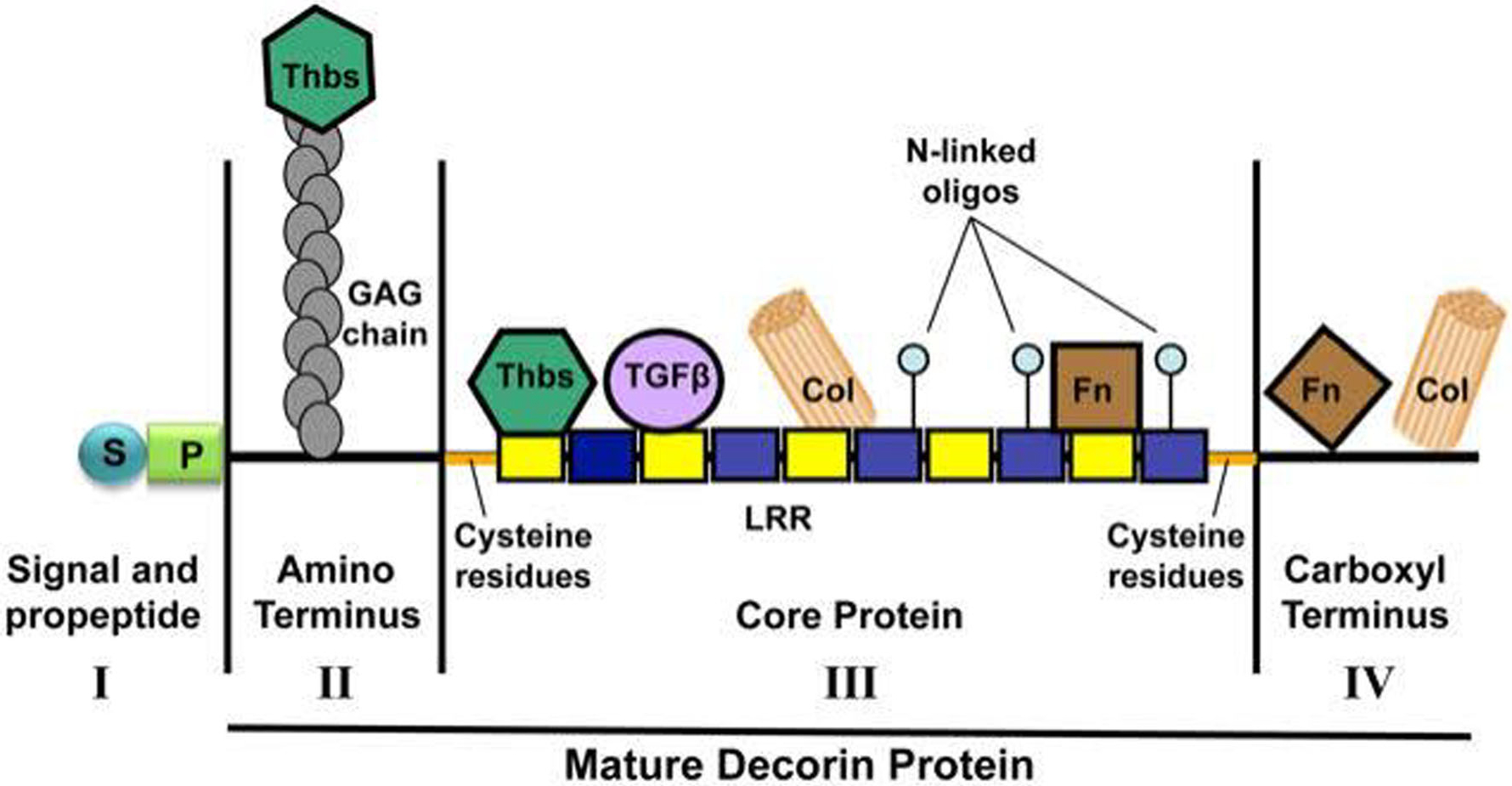
Schematic representation of the structural domains of the decorin protein. The four domains are labeled with Roman numerals. Domain I contains the signal peptide and propeptide, which are absent in the mature form of decorin. Domains II, III, and IV constitute the mature decorin protein. Domain II includes the amino terminus with the glycosaminoglycan (GAG) chain and has binding sites for thrombospondin (Thbs) and other GAGs. Domain III consists of the 40 kDa core protein with leucine-rich repeats (LRRs; shown as white and black boxes) characterized by the sequence LXXLXLXXNXL. This domain also has binding sites for collagen (high affinity), thrombospondin, TGF-β, and the heparin-binding region of fibronectin (Fn), along with N-linked oligosaccharides. Domain IV represents the carboxyl terminus, containing binding sites for fibronectin (cell-binding domain) and collagen (low affinity).

**FIGURE 2 |:**
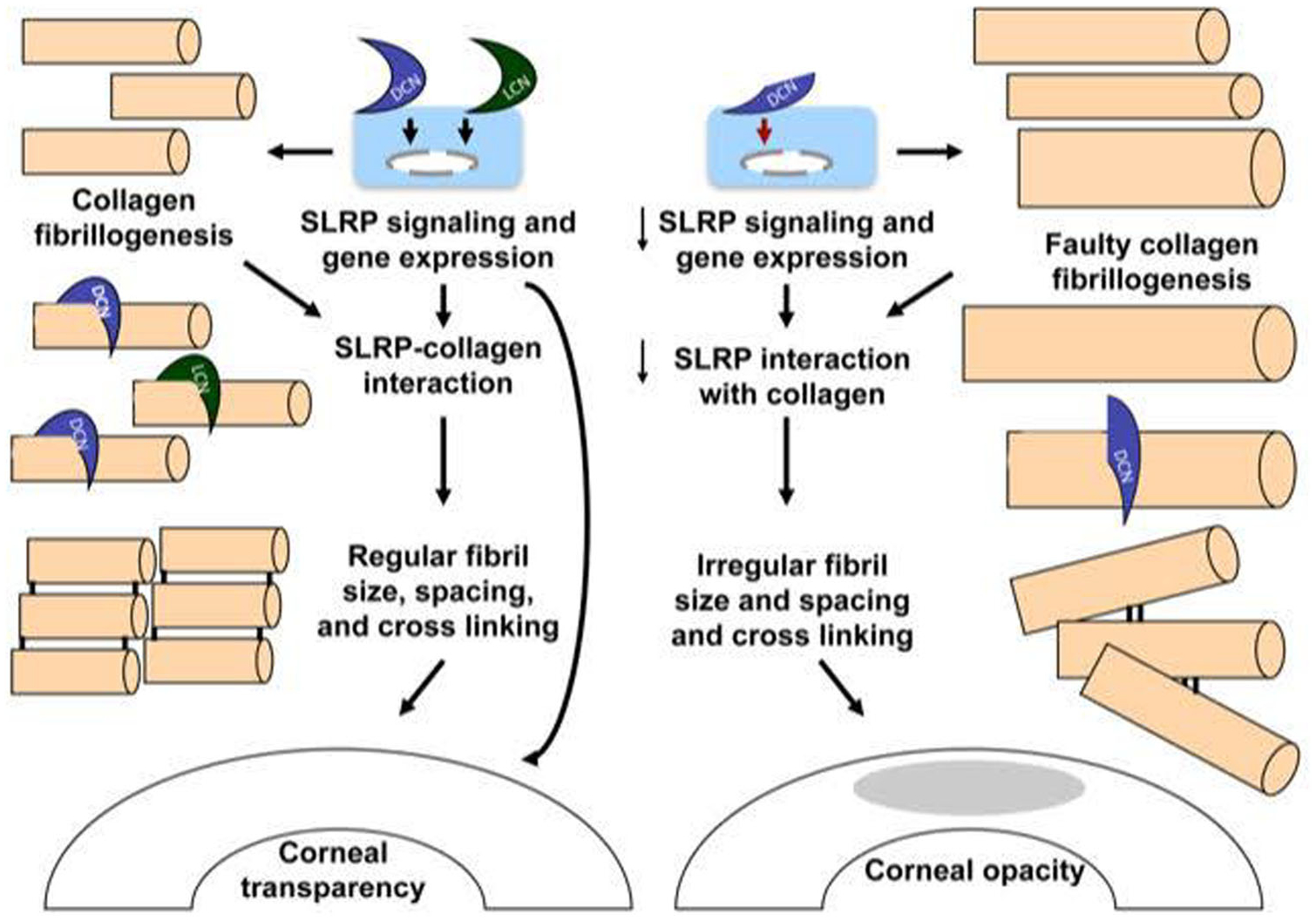
Schematic representation of corneal transparency and opacity through small leucine-rich proteoglycan (SLRP) and collagen interactions.

DCN, characterized by its core protein and attached glycosaminoglycan (GAG) chain, interacts with various ECM molecules, particularly collagen, and influences the organization and integrity of connective tissues. In the ocular surface, DCN’s role extends beyond mere structural support; it is involved in critical processes, such as collagen fibrillogenesis, modulation of growth factor activity, and regulation of cell behavior [4]. These functions are essential for maintaining corneal transparency, facilitating wound healing, and preventing fibrosis. However, the importance of DCN in ocular surface health is underscored by its involvement in several ocular surface disorders. Abnormal expression or dys-function of DCN has been implicated in conditions such as corneal scarring, pterygium [5], and keratoconus [6], highlighting its potential as a therapeutic target [7]. Hence, understanding the multifaceted roles of DCN in the ocular surface is crucial for developing novel therapeutic strategies aimed at treating or preventing these disorders. This review aims to provide a comprehensive overview of the current knowledge about DCN structure, expression, and function in the ocular surface [4]. It also explores the therapeutic potential of DCN-based treatments and discusses recent advancements and future directions in this field of research. Overall, this review seeks to elucidate the critical roles of DCN in ocular surface homeostasis and disease, thereby contributing to the development of innovative approaches for ocular surface therapy (Figures 1 and 2).

## 2 | Structure of DCN

DCN is a SLRP that consists of a core protein and a single GAG chain (Figure 1). It plays a crucial role in modulating the assembly and structural integrity of collagen fibrils in the ECM.

### 2.1 | Core Protein

The core protein of DCN is approximately 40 kDa in size and contains a series of leucine-rich repeats (LRRs). These LRRs are motifs that typically consist of 20–29 amino acids and are characterized by the presence of leucine residues at conserved positions. The LRRs in DCN form a curved, solenoid-like structure. This shape is stabilized by the repetition of leucine and other hydrophobic residues, creating a horseshoe-like appearance in three dimensions [1].

DCN contains 10–12 LRRs, which contribute to its ability to interact with other ECM components, particularly collagen. Each LRR forms a β-strand/α-helix structural unit, and these units stack together to create the curved structure. The inner concave surface of the LRRs is thought to be involved in protein-protein interactions, while the convex surface is less interactive but contributes to the overall stability of the structure [1].

### 2.2 | GAG Chain

DCN is decorated with a single dermatan sulfate (DS) or chondroitin sulfate (CS) GAG chain attached near the N-terminus of the core protein. This GAG chain extends outwards from the core protein and is highly hydrophilic. The GAG chain plays a significant role in binding to collagen fibrils and regulating their assembly. It contributes to the spacing between collagen fibrils, thus influencing the diameter and organization of the fibrils [4].

## 3 | Expression of DCN in the Ocular Surface

The ocular surface is a crucial entity of the eye, encompassing the cornea, conjunctiva, and tear film. Each component plays a vital role in maintaining ocular health, protecting the eye from external threats, and ensuring clear vision. A healthy ocular surface is essential for preserving the eye’s optical clarity, providing a barrier against pathogens, and facilitating wound healing. Disruptions to any part of this intricate system can lead to significant visual impairment and discomfort [1, 7].

### 3.1 | Cornea

DCN is predominantly located in the corneal stroma, where it is synthesized by corneal keratocytes (specialized fibroblasts residing in the stroma). DCN interacts with collagen fibrils, playing a crucial role in maintaining the organization and transparency of the corneal stroma. It regulates collagen fibrillogenesis, ensuring uniform spacing and alignment of collagen fibrils, which is essential for corneal clarity [1].

### 3.2 | Conjunctiva

Although specific expression patterns of DCN in the conjunctiva are less documented, it is known to be present in the conjunctival ECM. Its functions likely include modulating inflammation and facilitating wound healing, similar to its roles in the cornea and sclera [8].

### 3.3 | Sclera

DCN is detected throughout the scleral stroma, synthesized by scleral fibroblasts. It contributes to the structural integrity of the sclera by interacting with and regulating the assembly of collagen fibrils. DCN ensures the proper organization and spacing of collagen, which is vital for the biomechanical properties of the sclera [9].

## 4 | Biological Functions of DCN in the Ocular Surface

### 4.1 | Collagen Fibrillogenesis

DCN is crucial for collagen fibrillogenesis in the cornea and interacts with all major collagen-rich tissues, including type I and II collagen, co-localizing with large helical collagen fibers [4, 10]. The corneal stromal fibroblasts/keratocytes synthesize DCN within the ECM network [1]. It interacts with various growth factors and receptors, such as transforming growth factor beta (TGFB), epidermal growth factor receptor (EGFR), pigmented epithelium-derived factor (PEDF), and vascular endothelial growth factor (VEGF), regulating their biological activities [1, 8]. The GAG side chain of DCN can intermingle with stromal fibrils and serve as a reservoir for growth factors. DCN fills the interstitial spaces in the tissue microenvironment, facilitating ECM interactions by acting as a ligand for cell surface receptors like EGFR [11]. [12]. The role of DCN in regulating collagen fibrillogenesis is demontrated by Mohan et al [12] in a loss of function mouse model. DCN null (dcn−/−) mice exhibited an abnormal morphology and lower collagen levels with loose packing and asymmetrical organization compared to the wild type (dcn+/+), suggesting that DCN plays a role in regulating stromal fibrillogenesis and transparency during corneal healing [12]. Many such studies have been conducted before this, showing the role of DCN in corneal fibrillogenesis and ECM remodeling by conducting various in vitro and in vivo experiments [4, 10, 13]. The expression of DCN in ocular surface tissues is subject to regulation by various factors, including growth factors, cytokines, and mechanical stress. TGF-β, a key regulator of ECM components, has been shown to influence DCN expression [13]. Additionally, DCN itself can modulate the activity of growth factors such as TGF-β, creating a feedback loop that is crucial for maintaining tissue homeostasis [1, 14]. The study done by Mohan et al. [13] investigated the potential of DCN gene transfer to inhibit TGFβ-driven myofibroblast formation and corneal haze, conditions associated with corneal injuries [13]. Human corneal fibroblasts (HCF) were transfected with DCN cDNA using a mammalian expression vector. DCN significantly decreased TGFβ-induced alpha smooth muscle actin (αSMA) expression by 80–83% under serum-free conditions, compared to untransfected HCF without altering HCF phenotype or viability [13]. Additionally, DCN overexpression reduced RNA levels of fibronectin and collagen I, III, and IV, which are crucial for corneal wound healing. DCN gene transfer can effectively prevent TGFβ-driven transformation of keratocytes and corneal fibroblasts into myofibroblasts, offering a potential therapeutic approach for treating corneal haze in vivo. However, it is essential to recognize that DCN does not act alone in regulating collagen fibrillogenesis. It operates in concert with other SLRPs and ECM components, such as tenascin-X, lumican, and fibromodulin, which also play critical roles in collagen fiber spacing and structural organization [8, 15–17].

### 4.2 | Corneal Neovascularization (CNV)

**FIGURE 3 |:**
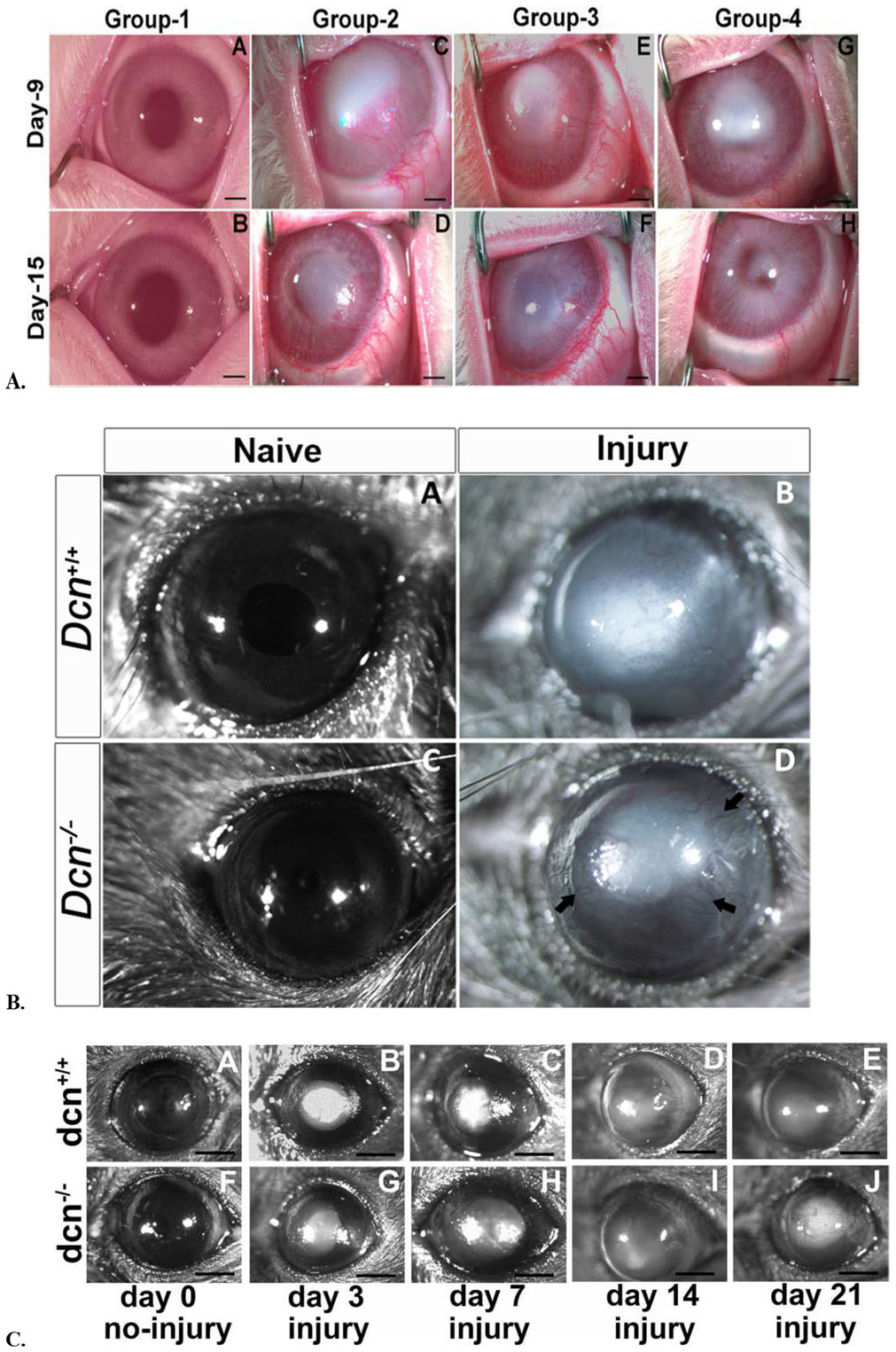
Experiments demonstrating the role of decorin in corneal fibrosis and neovascularization. (A) Representative stereo-biomicroscopic images displaying the impact of AAV5-DCN + AAV5-PEDF therapy on corneal fibrosis and corneal neovascularization (CNV) induced by chemical injury in a rabbit eye model across four different groups. Group 1 (naïve) shows healthy, untreated corneas with no signs of fibrosis or CNV (A, B). Group 2 (injury alone) represents eyes that underwent alkali injury, exhibiting severe fibrosis and CNV (C, D). Group 3 (no therapy) demonstrates corneas treated with an AAV-naked vector post-injury, showing similarly high levels of fibrosis and CNV as seen in Group 2 (E and F). Group 4 (therapy) features corneas treated with a combination of AAV5-DCN and AAV5-PEDF following injury, revealing markedly reduced fibrosis and CNV levels compared to both Group 2 and Group 3 on day 9 and day 15 (G and H). Clinical scores for haze (Fantes scale) and CNV (pixel intensity) are presented for all groups, confirming the therapeutic efficacy of the combined treatment in reducing pathological changes. Images captured at 7.1x magnification. Scale bar = 2 mm. (B) Representative stereo-microscopic images illustrating the role of decorin in preventing corneal neovascularization (CNV) in a murine alkali injury model. Images of wild-type (dcn+/+) and decorin-deficient (dcn−/−) mice corneas are shown before injury and 21 days post-alkali injury. In the Dcn+/+ mice, minimal CNV is observed 21 days post-injury, indicating decorin’s protective role in maintaining corneal avascularity (A, B). In contrast, dcn−/− mice exhibit significantly increased CNV at day 21 post-injury, highlighting the critical function of decorin in modulating angiogenic responses following corneal damage (C, D). (C) Representative slit lamp images showing the role of decorin in corneal clarity and neovascularization following alkali injury in a murine model. Uninjured corneas from both wild-type (dcn+/+) and decorin gene-deficient (dcn−/−) mice appear clear and free of neovascularization (A, F). Post-alkali injury, both groups develop corneal haze and neovascularization, with more severe effects observed in the dcn−/− mice. By Day 21, dcn−/− corneas display significantly greater haze and neovascularization compared to the dcn+/+ corneas (E vs. J), demonstrating decorin’s role in mitigating these effects.

DCN plays a significant role in regulating CNV [18–20]. The DCN’s interaction with VEGF and TGF-β1 are crucial factors in CNV [18]. The cleavage of DCN by membrane type 1-matrix metalloproteinase (MT1-MMP) facilitates angiogenesis by freeing up pro-angiogenic factors and altering ECM structure, emphasizing the importance of ECM remodeling in CNV [21]. DCN also regulates the function of FN1, an ECM protein upregulated in vascularized corneas that promotes angiogenesis and endothelial cell proliferation [19]. Although a direct interaction with tenascin-C (TNC) is not detailed, DCN’s regulation of collagen and FN1 influences the overall ECM dynamics, where TNC also promotes vascularization. By maintaining collagen organization, DCN contributes to corneal stiffness, and its reduced expression in vascularized corneas leads to decreased tissue rigidity, facilitating vascular invasion. Additionally, DCN has an anti-angiogenic role, retarding CNV by downregulating pro-angiogenic factors like VEGF and angiopoietin. Mutations or deletions in the DCN gene can lead to corneal dystrophies, underscoring its importance in preventing abnormal vascularization and maintaining corneal health [20]. Additionally, DCN interacts with other angiogenic factors, such as fibroblast growth factor (FGF) and platelet-derived growth factor (PDGF), preventing excessive neovascularization and preserving corneal transparency [19]. Figure 3A shows the effect of AAV5-DCN and AAV5-PEDG combination therapy on the in vivo model [22]. Collectively, these findings elucidate the multifaceted role of DCN in CNV, highlighting its ability to modulate key angiogenic pathways and maintain corneal avascularity through complex interactions with various growth factors and ECM components [19]. By modulating VEGF activity, DCN not only controls neovascularization but also influences the infiltration and behavior of immune cells at the site of injury [23].

### 4.3 | Immune Cell Regulation, Inflammation, and Neuroprotection

DCN plays a key role in corneal inflammation and wound healing by interacting with inflammatory cytokines and growth factors, thereby modulating the immunological landscape of the cornea and facilitating a balanced healing process [2, 8, 24]. It exerts a significant influence by binding to and inhibiting key inflammatory mediators, such as tumor necrosis factor-alpha (TNF-α) and interleukin-1beta (IL-1β), crucial cytokines in the inflammatory response. This inhibition helps reduce acute inflammatory reactions and prevents chronic inflammation that can lead to tissue damage [2, 24, 25]. DCN’s impact on the immune system extends to its role in immune cell regulation; it affects macrophage activation and the balance of M1/M2 macrophage phenotypes. By influencing these macrophage states, DCN can shift the inflammatory environment from a pro-inflammatory (M1) to a tissue-regenerating (M2) state, which is crucial for healing and resolution of inflammation [23, 24]. This proteoglycan influences neuroprotection through its ability to modulate the ECM and interact with growth factors that are critical for nerve health [26]. DCN enhances nerve regeneration by stabilizing the microenvironment around corneal nerves, supporting neuronal growth and survival. This effect is partly mediated by its interaction with nerve growth factor (NGF), which is essential for the maintenance and repair of neuronal tissues [23]. By promoting a supportive milieu for nerve fibers, DCN helps maintain the sensory and reflex functions of the cornea, which are crucial for eye health and visual acuity. These neuroprotective attributes highlight DCN’s potential as a therapeutic agent in treating corneal conditions characterized by nerve impairment [23, 25, 26].

## 5 | DCN in Ocular Surface Homeostasis and Wound Healing

DCN plays a pivotal role in corneal wound healing by regulating collagen fibrillogenesis, by regulating the spatial arrangement and uniform diameter of collagen fibrils within the ECM. DCN binds to collagen type I fibrils, influencing fibril maturation and spacing, which is essential for the biomechanical properties and optical clarity of the cornea [12]. In vivo studies using DCN-deficient mice reveal disorganized collagen networks with irregular fibril diameters, leading to altered stromal architecture and impaired corneal transparency. This highlights the critical role of DCN in maintaining ECM integrity and preventing fibrosis during corneal wound healing by precisely modulating collagen fibril assembly and organization [12]. This regulation is essential for restoring corneal transparency and function post-injury, as shown in Figure 3C [12].

In addition to collagen regulation, DCN significantly impacts corneal re-epithelialization and haze reduction. Research involving an ex vivo wound healing model with equine amniotic membrane suspension demonstrated that DCN enhances re-epithelialization and minimizes corneal haze formation, a common complication in wound healing that affects visual acuity [27]. During corneal stromal wound healing, major regulators like TGF-β and FGF interact with DCN [28]. These interactions help promote controlled healing and prevent excessive scarring, highlighting DCN’s importance in managing wound healing dynamics [3]. Dynamic changes in the ECM are a hallmark of corneal wound healing [3]. DCN helps maintain ECM homeostasis and integrity during this process. Following corneal alkali burns in mice, DCN is involved in ECM deposition and remodeling, which are crucial for effective wound healing and minimizing fibrosis [29]. The modulation of ECM components by DCN ensures that the corneal structure is restored without excessive scarring [29]. DCN’s role extends beyond the cornea, influencing other ocular tissues and diseases [7]. Research has indicated that DCN regulates cellular behavior and extracellular interactions in the lens and other parts of the eye, demonstrating its broader significance in ocular health [28]. An innovative therapeutic approach involves the sustained release of DCN to the eye’s surface. This method has shown promise in enabling scarless corneal regeneration by modulating ECM components and cellular responses, thus providing a controlled healing environment and reducing the likelihood of fibrosis [3, 28, 29]. The disorganization of the transparent collagenous matrix in the cornea, often resulting from various infections and inflammatory conditions, can lead to corneal opacity and subsequent sight loss. In a study using a murine model of Pseudomonas keratitis, the application of this novel eye drop resulted in significant reductions in corneal opacity within 16 days. More notably, the addition of human recombinant DCN (hrDCN) led to the restoration of corneal epithelial integrity with minimal stromal opacity. This improvement was endorsed by reduced levels of αSMA, fibronectin, and laminin, all markers of fibrosis and scarring. The sustained delivery of DCN through this innovative eye drop formulation holds great promise as a non-invasive anti-fibrotic treatment for patients with microbial keratitis. This treatment approach potentially eliminates the need for surgical intervention, offering a sight-saving solution for many individuals, particularly in the developing world, where access to corneal transplantation may be limited. Thus, DCN’s healing effects, facilitated by this advanced drug delivery system, represent a significant breakthrough in the management of corneal scarring and opacity [30].

DCN modulates corneal wound healing by binding to the EGFR on corneal fibroblasts. This interaction triggers caveolae-mediated endocytosis, leading to EGFR internalization and subsequent downregulation of its downstream signaling pathways, including the ERK1/2 pathway. As a result, the migratory capacity of corneal fibroblasts is reduced, preventing excessive cell movement and limiting the risk of fibrosis. Through this mechanism, DCN ensures a controlled and balanced wound healing response, maintaining corneal transparency and preventing scarring [11].

## 6 | DCN in Ocular Surface Diseases

Studies on congenital stromal corneal dystrophy (CSCD) highlight the significant impact of mutations in the DCN (DCN) gene. A single base pair deletion (c.941delC) in the coding sequence of the DCN gene, predicting a C-terminal truncation of the decorin protein (p.Pro314fsX14), was identified in two individuals from a family with CSCD [31]. Further research demonstrated that DCN accumulation in the corneal stroma leads to the characteristic opacities of CSCD. The retention of truncated DCN within the cells causes endoplasmic reticulum stress and unfolded protein response, providing a mechanism behind the disease [32]. Novel mutations in the DCN gene were found in both (c.962delA) Chinese [33] and (p.His317Thrfs*11) Armenian [34] families with CSCD, expanding the genetic heterogeneity of the condition. A pediatric case involving a novel DCN variant (c.953del) further illustrated the clinical variability of CSCD [35]. Comprehensive clinical, genetic, and histological analyses in Spanish families emphasized the importance of detailed phenotypic and genotypic characterization [36]. Research into the role of the DCN core protein in collagen organization revealed that mutations disrupt its interaction with collagen, resulting in disorganized collagen fibrils [37]. Collectively, these studies accentuate the critical role of DCN in congenital diseases, particularly in maintaining corneal transparency and structure, and highlight the diverse genetic mutations in CSCD [38]. The role of ECM remodeling is well known in the cornea.

Pterygium is a fibrovascular growth of the conjunctiva onto the cornea, driven by UV-induced oxidative stress, chronic inflammation, and dysregulated ECM remodeling. DCN is considerably downregulated in the pterygium compared to healthy ones [5]. This shows a potential role of DCN in various ocular surface diseases [5, 6].

**FIGURE 4 |:**
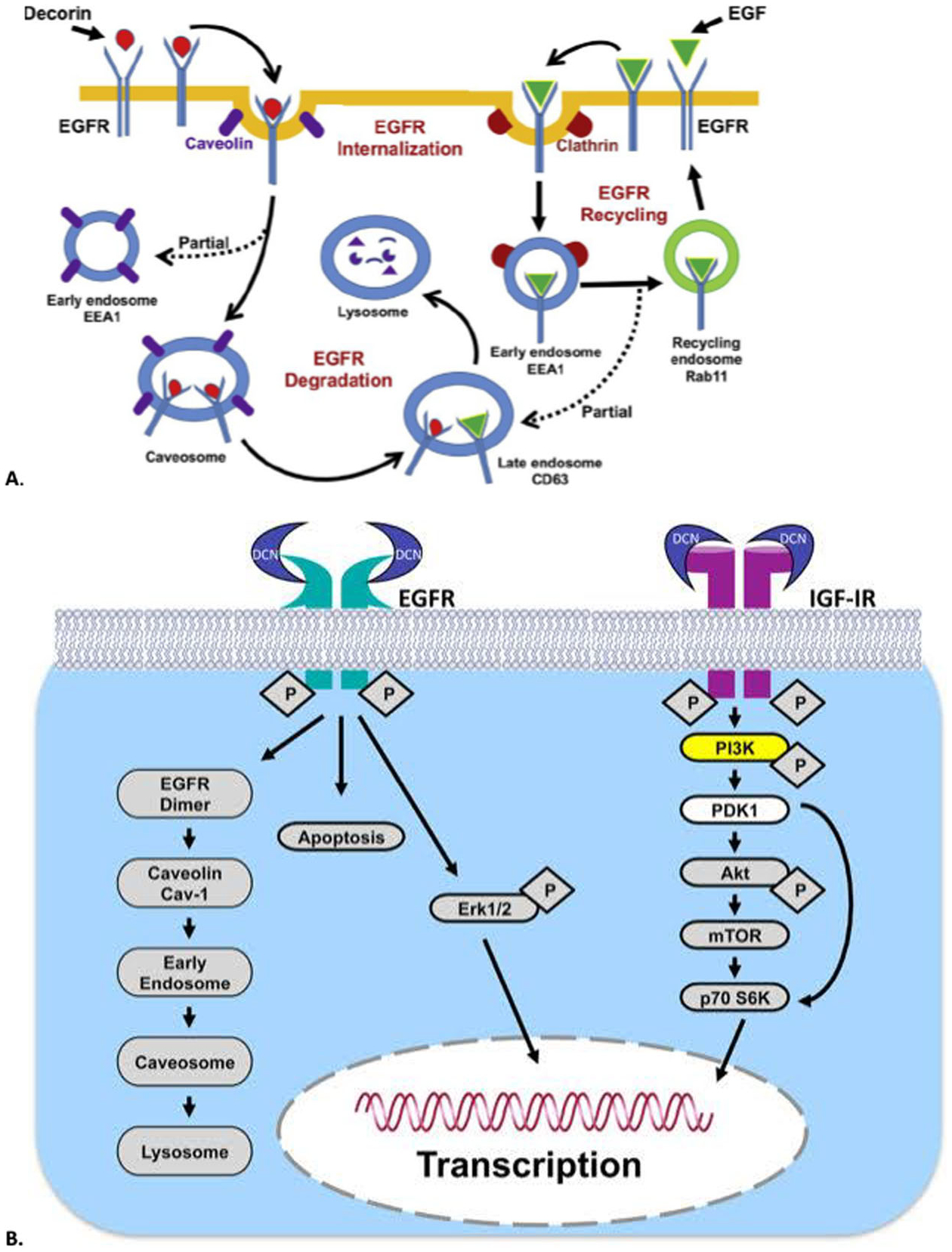
(A) Schematic representation illustrating the distinct internalization pathways of EGFR mediated by decorin and EGF. Decorin binds to EGFR, forming a DCN-EGFR complex, which is internalized and bypasses the early endosome, being directly transferred to perinuclear vesicles (caveosomes). The DCN-EGFR complex is subsequently routed to the late endosome (CD63) for degradation. In contrast, EGF triggers stepwise endocytosis of EGFR, primarily through early endosomes (EEA1). From there, the EGF-EGFR complex is either recycled via the recycling endosome (Rab11) or directed to the late endosome (CD63) for degradation. This schematic highlights the differential regulation of EGFR internalization by decorin and EGF, emphasizing decorin’s unique pathway that bypasses the early endosome. (B) Representative image showing decorin’s involvement in EGFR and IGF-IR signaling pathways. Decorin binds to EGFR, triggering its phosphorylation and leading to distinct signaling outcomes, including EGFR dimerization, caveolin-mediated internalization, early endosome and caveosome formation, and subsequent lysosomal degradation. Decorin-induced EGFR phosphorylation also activates Erk1/2, promoting target gene transcription (e.g., p21), and induces apoptosis through a caspase-dependent pathway. Additionally, decorin interacts with IGF-IR, activating the PI3K pathway via PDK1, Akt, mTOR, and p70 S6K, driving target gene expression. PI3K is also involved in the TGF-β signaling pathway via decorin regulation through LRP1, and decorin-mediated PI3K/Akt signaling may reduce apoptosis.

**FIGURE 5 |:**
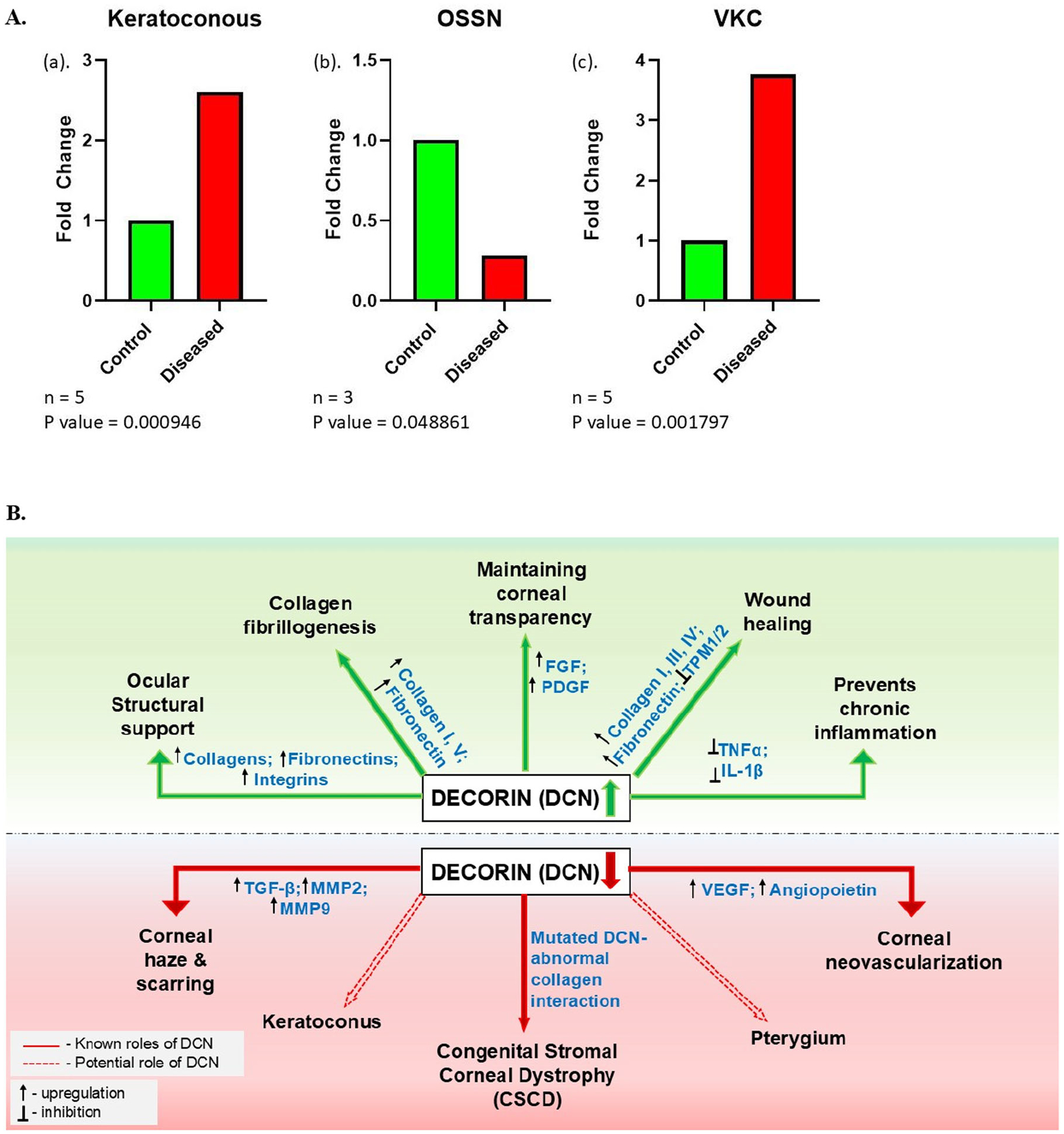
(A) Expression of DCN in different ocular surface diseases (a) keratoconus: expression of DCN in the corneal epithelium of keratoconic eyes compared to healthy corneal epithelium. A significant upregulation of DCN expression was observed with a fold change of 2.609 (*n* = 5, *p* = 0.000946). (b) Ocular surface squamous neoplasia (OSSN): Expression of DCN in OSSN tumors compared to healthy conjunctiva. A significant downregulation of DCN expression was found with a fold change of 0.284 (*n* = 3, *p* = 0.04886). (c) Vernal Keratoconjunctivitis (VKC): Expression of DCN in the diseased conjunctiva of VKC patients compared to healthy conjunctiva. DCN expression was significantly upregulated with a fold change of 3.763 (*n* = 4, *p* = 0.001797). (B) The role of decorin in corneal homeostasis and pathology. Decorin regulates collagen fibrillogenesis, maintaining proper stromal organization and transparency. It modulates wound healing by controlling cell proliferation and migration, preventing excessive fibrosis. Decorin also interacts with growth factors, such as TGF-β, to limit pro-fibrotic signaling, and plays an anti-inflammatory role by reducing immune cell infiltration. Dysregulation of decorin leads to disrupted collagen organization, fibrosis, and inflammatory damage, contributing to corneal opacity and scarring.

Keratoconus is characterized by progressive corneal thinning and ectasia driven by ECM remodeling, oxidative stress, and inflammatory signaling. Although the role and expression of DCN are not well studied in its pathophysiology, it is obvious that it plays a role in it [39]. Expression of DCN was analyzed in various ocular surface diseases using quantitative real-time PCR (qRT-PCR). In the corneal epithelium of keratoconus patients (*n* = 5), DCN expression was significantly upregulated with a fold change of 2.609 compared to healthy corneal epithelium (*p* = 0.000946). This suggests that DCN may play a crucial role in the pathophysiology of corneal ECM remodeling, a key factor in keratoconus. In this disease, ECM remodeling leads to a weakened cornea, contributing to its structural instability and increased intraocular pressure (IOP) (Figures 4 and 5).

Similarly, DCN expression was evaluated in the conjunctiva of severe vernal keratoconjunctivitis (VKC) patients (*n* = 4), where it was found to be 3.763-fold upregulated compared to healthy conjunctiva (*p* = 0.001797). This is the first report of DCN expression in the conjunctiva of VKC patients, suggesting a potential role in conjunctival ECM remodeling. This upregulation may contribute to CNV and pannus formation, which are common features in severe cases of VKC, though the precise mechanisms remain to be fully explored.

In ocular surface squamous neoplasia (OSSN), a surface cancer of the eye, DCN expression was significantly downregulated. Analysis of OSSN tumor samples (*n* = 3) compared to healthy conjunctiva revealed a fold change of 0.284 (*p* = 0.04886). Given that DCN is known for its tumor-suppressive properties in various cancers, its downregulation in OSSN correlates with tumor progression, highlighting its potential role in inhibiting tumor growth when expressed at normal levels [40–42]. Taken together, these findings demonstrate that DCN has a crucial role in the pathophysiology of various ocular surface diseases. Its involvement in ECM remodeling and tumor suppression suggests a variety of functions that warrant further exploration, providing valuable insights into the underlying mechanisms of these diseases.

## 7 | Therapeutic Potential of DCN in Ocular Surface Disorders

DCN has demonstrated significant therapeutic potential in treating ocular surface conditions, with its delivery methods playing a crucial role in its efficacy. These methods range from topical applications to advanced gene therapy techniques, each tailored to maximize therapeutic outcomes for specific ocular conditions.

- Topical Applications: Topical formulations of DCN have been extensively used to address corneal inflammation and promote neuroprotection. It was demonstrated in mouse models of benzalkonium chloride-induced corneal neuropathy [25]. This approach allows for direct, localized treatment of the cornea, facilitating rapid intervention that is easy to administer. Similarly, the effect of topical DCN on temporal changes to corneal immune cells after epithelial abrasion highlights its role in modulating local immune responses, enhancing the healing process [23, 25, 26, 43]. Furthermore, topical DCN treatment has been shown to promote corneal epithelial nerve regeneration after abrasion injury in Cx3cr1-deficient mice, which lack resident corneal epithelial dendritic cells. These results suggest that DCN induces corneal nerve regeneration through the activation of dendritic cells. Advantages of this method include ease of use, non-invasiveness, and its ability to provide targeted action with minimal systemic side effects. However, its limitations include the potential for limited retention time on the ocular surface, requiring frequent administration, and reduced efficacy in severe or chronic conditions that require prolonged exposure to therapeutic agents [7, 26]
- Sustained Release Systems: The sustained release of DCN directly to the ocular surface has been shown to enable scarless corneal regeneration. These systems are specifically designed to overcome the limitations of traditional eye drop formulations, which often have short retention times due to blinking and tear drainage. By employing materials such as viscoelastic polymers or gel-forming carriers, these systems ensure a continuous supply of DCN at the site of injury or disease, maintaining therapeutic levels over extended periods. Such systems can be particularly advantageous for chronic conditions or where long-term treatment is necessary to prevent complications like scarring. This approach allows for less frequent dosing, improving patient adherence while ensuring a consistent supply of the drug at therapeutic levels to enhance treatment effectiveness. Furthermore, these systems address the challenge of delivering anti-scarring therapies effectively in the dynamic environment of the ocular surface. However, challenges such as designing complex systems, potential inconsistencies in drug release rates, and the necessity of using biocompatible materials to avoid adverse effects remain key limitations [32].
- Gene Therapy Approaches: Gene therapy represents a cutting-edge delivery method for DCN, utilizing vectors such as adeno-associated virus (AAV) to achieve targeted and sustained expression. AAV-mediated DCN delivery has been employed to retard CNV and to inhibit corneal scarring significantly [22, 44], with a 6-month in vivo safety profiling confirming its long-term safety and efficacy [43]. Moreover, combination gene therapy approaches using AAV5-DCN and AAV5-PEDF have been developed to tackle both fibrosis and neovascularization concurrently, offering a comprehensive treatment strategy for complex corneal diseases. This method offers long-term therapeutic effects, precise targeting, and the capability to address multiple disease mechanisms at once. However, it is associated with high costs, a risk of immune reactions, and significant technical challenges in development and delivery [22, 43–46].
- Amniotic Membrane Suspensions: Amniotic membrane suspension enriched with DCN offers an innovative approach in reducing haze by promoting corneal re-epithelialization in equine wound healing models. This method leverages the natural healing properties of amniotic membrane combined with the bioactive benefits of DCN, providing a synergistic effect that accelerates healing and reduces visual impairment following injury. This approach is valued for its bio-compatibility, natural origin, and capacity to create a supportive environment for regeneration. However, challenges include variability in the quality of amniotic membrane preparations, restricted availability, and the requirement for specialized storage and handling [27].

Each of these delivery methods harnesses the unique properties of DCN to address different aspects of ocular surface pathology. Whether through direct application, sustained release, or genetic modulation, the strategic delivery of DCN has opened new avenues for the treatment of corneal diseases, enhancing both efficacy and patient outcomes in ocular therapy [8, 28, 47, 48].

## 8 | Current Research

The summarized table provides a comprehensive overview of the multifaceted roles of DCN in the ocular surface, delineating its impact from basic research through potential clinical applications. From scarless corneal regeneration and anti-inflammatory actions to its contributions in wound healing and anti-neovascularization, the research reinforce DCN’s pivotal role in improving ocular health. The studies utilize a range of models, from animal experiments to advanced gene therapy approaches, demonstrating DCN’s versatility and its potential to address a spectrum of ocular conditions (Table 1).

## 9 | Future Directions

The future of DCN in the ocular surface holds exciting prospects, with several technical and clinical pathways to explore that could significantly advance its therapeutic applications.

- Advanced Delivery Mechanisms: One of the crucial aspects of bringing DCN-based therapies to market involves refining delivery mechanisms. Innovations such as nanoparticle carriers, hydrogel systems, and biodegradable implants could enhance the bioavailability and sustained release of DCN at targeted ocular sites. These technologies promise to improve treatment efficacy and patient compliance by providing more precise dosing and reducing the frequency of administration.
- Gene Therapy and Genetic Editing: Advances in gene therapy, particularly using AAV vectors, offer powerful methods to deliver DCN directly to ocular tissues, potentially correcting genetic defects at their source. CRISPR/Cas9 and other gene-editing technologies could be employed to modify or correct mutations in the DCN gene associated with congenital ocular conditions. These genetic strategies not only aim to treat existing damage but also prevent the onset of disease symptoms, shifting the focus from symptomatic treatment to preventive care.
- Clinical Trials and Regulatory Pathways: To translate the benefits of DCN from the laboratory to clinical settings, comprehensive clinical trials are essential. Studies must demonstrate the safety, efficacy, and consistency of DCN-based treatments across diverse patient populations. Establishing robust regulatory pathways will also be critical to ensure that these new therapies meet stringent health standards and gain approval for use in clinical practice.
- Expanding Research into Other Ocular Surfaces: Beyond the cornea, exploring DCN’s role in diseases affecting other parts of the ocular surface, such as the conjunctiva and the lacrimal system, could provide new therapeutic avenues. DCN’s involvement in fibrosis, wound healing, and immune regulation suggests its potential utility in conditions like pterygium, dry eye disease, and ocular cicatricial pemphigoid.
- Interdisciplinary Collaboration: The complexity of translating a molecular therapy into a viable clinical treatment requires interdisciplinary collaboration among molecular biologists, pharmacologists, clinicians, and regulatory experts. This collaborative effort is crucial for navigating the challenges of drug development, from synthesis and formulation to clinical trials and market approval. As research on DCN continues to evolve, these technical, clinical, and collaborative efforts will be essential to realize the full potential of DCN as a transformative therapy for ocular surface diseases. This comprehensive approach not only aims to bring effective treatments to patients sooner but also opens the door to discovering new roles for DCN in ocular health and disease.

## 10 | Conclusion

DCN’s integral role in ocular surface health and disease is evident through its involvement in key physiological processes, such as collagen fibrillogenesis, inflammation modulation, and corneal wound healing. As a vital component in maintaining ocular clarity and functionality, the therapeutic potential of DCN extends from preventing fibrosis to enabling scarless healing and mitigating pathological neovascularization. While significant strides have been made in understanding DCN’s role in the cornea and sclera, its presence and function in the conjunctiva remain less explored. Investigating DCN’s involvement in conditions such as conjunctivitis and Stevens-Johnson syndrome (SJS) could reveal new therapeutic avenues, emphasizing the need for broader research into its role across various ocular surface diseases. Furthermore, the protective role of DCN in ocular health is well recognized, but identifying and studying the regulators of DCN expression and activity could provide deeper insights into its complex biological functions. Such studies could enhance our ability to manipulate DCN levels and activity for therapeutic benefits.

DCN expression is upregulated in keratoconus and VKC, contributing to ECM remodeling and potential neovascularization, while its downregulation in OSSN aligns with its tumor-suppressive role, highlighting its therapeutic potential across ocular diseases. The comprehensive understanding and application of DCN-based therapies could significantly transform the landscape of ocular medicine, providing improved management and treatment options for a myriad of ocular conditions.

In terms of clinical applications, the potential for using gene therapy to address conditions like CSCD showcases DCN’s utility in genetically based interventions. This approach could lead to novel treatments that directly correct the underlying genetic anomalies affecting DCN function. Given DCN’s wide range of targets and its critical role in various key processes of ocular health, it may well be considered an ultimate therapeutic molecule. As research continues to unfold, DCN’s profound impact on ocular surface health and disease highlights its potential as a cornerstone of future therapeutic developments.

## Figures and Tables

**TABLE 1 |:** Current studies compiling the role of DCN in ocular surface.

Research focus	Model and therapy	Key findings	Future directions	References
Scarless corneal regeneration	Murine model of *Pseudomonas keratitis with topical treatment*	Sustained release of DCN promotes scarless regeneration and prevents fibrosis, maintaining corneal transparency.	Explore long-term outcomes and optimize vector delivery systems for clinical translation.	[30]
Anti-inflammation and neuroprotection	Mouse models of benzalkonium chloride-induced corneal neuropathy and treatment with topical DCN	Topical DCN reduces inflammation and supports neuroprotection, particularly effective in chemical-induced corneal injuries.	Conduct clinical trials to evaluate efficacy and safety in humans; develop sustained release formulations.	[25]
Wound healing	*Mouse corneal alkali injury model, with gene knockout*	DCN enhances corneal wound healing by regulating collagen fibrillogenesis, improving epithelial recovery, and reducing haze formation.	Investigate the role of DCN in modulating other extracellular matrix components during the healing process.	[12]
Anti-neovascularization	Rabbit and mouse alkali injury for neovascularization using AAV-mediated DCN delivery.	DCN gene therapy effectively reduces neovascularization, crucial for maintaining clear vision by controlling pathological angiogenesis.	Assess long-term effects and immune responses; explore combination therapies with other anti-angiogenic factors.	[19, 22, 46]
Immune modulation	C57BL/6 J mice models on epithelial abrasion with topical treatment.	DCN impacts corneal immune cell dynamics, modifying cytokine profiles to facilitate the resolution of inflammation and tissue repair.	Study the interaction of DCN with specific immune cell types and cytokine pathways to develop targeted immunomodulatory therapies.	[23, 24]
Comprehensive ocular therapy	Rabbit model of fibrosis and neovascularization with combined gene therapy.	Combination gene therapy using DCN and other factors targets multiple pathological pathways, offering a holistic treatment approach.	Investigate the synergistic effects of DCN with other therapeutic molecules; evaluate potential for treating a broader range of ocular surface diseases.	[22]
Congenital effects	Genetic studies and clinical case analyses in human subjects.	Mutations in the DCN gene are associated with congenital stromal corneal dystrophy, affecting collagen fibril formation and leading to visual impairment.	Explore gene therapy and advanced genetic editing techniques to correct or mitigate the effects of harmful mutations in DCN associated with congenital disorders.	[36]

## Data Availability

The data can be made available on request.
